# Obesity education for front-line healthcare providers

**DOI:** 10.1186/s12909-018-1380-2

**Published:** 2018-11-23

**Authors:** Diana C. Sanchez-Ramirez, Heather Long, Stephanie Mowat, Casey Hein

**Affiliations:** 1 Office of Continuing Competency and Assessment, Rady Faculty of Health Sciences, University of Manitoba, 260-Brodie Centre, 727 McDermot Avenue, Winnipeg, MB R3E 3P5 Canada; 2 Office of Educational and Faculty Development, Rady Faculty of Health Sciences, University of Manitoba, Winnipeg, Canada; 3 Department of Psychology, University of Western Ontario, London, Canada; 4 College of Dentistry, Rady Faculty of Health Sciences, University of Manitoba, Winnipeg, Canada

**Keywords:** Obesity, Interprofessional education, Healthcare

## Abstract

**Background:**

To assess the effect of an interprofessional educational activity on professional skills, attitudes, and perceived challenges toward obesity management among front-line healthcare providers.

**Methods:**

A one-day interprofessional obesity education activity was organized for healthcare providers across various disciplines. All participants were invited to complete an anonymous survey pre- and post-event, and at six-month post-event. The survey was created based on a comprehensive list of perceived skills, professional attitudes and challenges toward obesity intervention compiled from existing literature.

**Results:**

Sixty-seven healthcare providers completed the survey pre- and post-event. Participants reported increases in professional *skills* such as their ability to assess weight (*p* = 0.04), to address weight management issues (*p* < 0.001), to teach/motivate patients toward physical activity (*p* < 0.001) and healthy eating practices (*p* = 0.001), to use behavior modification techniques (*p* < 0.001), and to deal with family issues (*p* < 0.001). Professional *attitudes*: practitioners felt more educated/competent in obesity management (*P* < 0.001), learned where to refer patients (*p* < 0.001), were more comfortable in discussing obesity in managing obesity (*p* < 0.001), were less likely to avoid the topic (*p* = 0.004) and felt less frustrated with the low success rate (*p* = 0.030). Enhancement in *professional attitudes* remained 6 months after the event. Improvements were mainly associated with male gender, younger age, fewer years of professional practice and healthcare professionals other than physicians. No statistically significant changes in perceived *challenges* were found after the educational event.

**Conclusion:**

Results of this study showed that this interprofessional learning activity contributed to the improvement of professional skills and attitudes of front-line healthcare providers caring for those who are obese or at risk for obesity. The positive results of this interprofessional learning activity aligns with the training needs identified by healthcare practitioners in previous studies, and suggest that this design and content could be used to guide future educational programming in the care of obese people.

## Background

Worldwide obesity has nearly tripled since 1975 reaching more than 1.9 billion (39%) adults aged 18 years and over, and ~ 340 million (18%) children and adolescents in 2016 [1]. In the same year, overweight and obesity affected an estimated of 68% of adults in USA, as well as 64% of adults and 32% of children and adolescents in Canada [2]. Although largely preventable, obesity remains a proven major risk factor for a wide array of non-communicable diseases such as heart disease, stroke, diabetes, osteoarthritis and some forms of cancer [3]. Therefore, the need to find effective obesity prevention and intervention strategies has become one of the most profound challenges in public health.

Several psychological theories have been use to promote health behavior change (HBC) in the prevention and management of patients with various health conditions including obesity [4]. While behavioral interventions seem to be effective in promoting weight loss, weight loss maintenance is still a challenge. However, there is some support for the effectiveness of HBC interventions based on self-determination and self-regulation theories in long-term weight management [4–6]. A recent review of the evidence indicates that the combination of cognitive behavioral therapy, personalised diet and exercise is the most effective form of intervention in overweight and obesity [7].

Obesity is a complex systems problem [8], where individual behaviors related to nutrition and physical activity interact with genetics, socioeconomic factors (e.g. access to healthy foods), awareness of best nutrition and exercise practices, and so on, making obesity a very complicated issue for healthcare providers to manage in their practices [9, 10]. Previous studies have explored obesity management among primary care providers and identified important challenges in the treatment of obesity [11–23]. The main practitioner-related challenges identified were lack of knowledge and training in aspects such as current assessment and counseling strategies, and behavior management techniques in obesity [12, 13]. Lack of patient/family involvement and motivation were also acknowledged [16]. Additional challenges identified included absence of reimbursement arrangement, referral options, consultation time and support services [19–21].

The complexity and multifactorial nature of obesity together with many other challenges could lead healthcare providers to feel overwhelmed and ineffective in their ability to influence this ever-increasing obesity epidemic [14, 17, 24]. In addition, some healthcare providers choose to avoid the issue altogether because they are uncomfortable about discussing overweight and obesity with their patients [12], and others feel that obesity is more of a public health matter and that there are simply not enough resources to deal with this issue [17]. Consequently, evidence has shown that rates of counseling for weight, diet and exercise in primary care have declined despite the increased rates of overweight and obesity in the USA [25].

Obesity is a significant threat to health that needs to be consistently addressed across all health disciplines. Evidence has shown that interprofessional teams improve obesity outcomes [26], encouraging healthcare providers to work cooperatively with people from other professions and disciplines to provide comprehensive care [27]. Therefore, as a means of responding to previously identified challenges to obesity prevention and intervention, an educational activity was organized to bring together a wide array of front-line healthcare professionals to discuss and partake in an interprofessional learning activity. The objective of this study was to assess the effect of the interprofessional educational activity on professional skills, attitudes, and perceived challenges toward obesity management among front-line healthcare providers from diverse healthcare disciplines.

## Methods

According with the literature, interprofessional education occurs when two or more professions learn about, from and with each other to enable effective collaboration and improve health/well- being (or both) of patients/clients [27, 28]. The Division of Continuing Competency and Assessment in the Rady Faculty of Health Sciences of the University of Manitoba hosted a full day, interprofessional educational activity entitled “*Obesity Intervention for Front-line Healthcare Providers*” (May 1, 2015).

This one-day accredited educational activity was planned by a committee composed of twelve professionals from diverse fields (dental hygiene, dentistry, nursing, pharmacy, nutrition, medicine, kinesiology, physical therapy and health education), who defined the most suitable themes, speakers, methods and format of assessment based on their expertise and informed by literature. It aimed to bring together a wide array of front-line healthcare providers including physicians, physician assistants, nurse practitioners, pharmacists, dentists, dental hygienists, dietitians, nutritionists, and social workers to participate in an interprofessional learning activity aimed to assist healthcare providers in caring for people who are obese or at risk for obesity. During this two-session program, leaders from our own professional communities presented on their experiences in caring for obese patients in different healthcare fields, and offered ideas for incorporating obesity intervention into everyday patient care. Furthermore, discussion of obesity-related cases during round table sessions with members of two or more professions was encouraged. All the participants were invited to complete an anonymous surveys (3 times) pre- and post-event, and at six-months. This study was approved by the Health Research Ethics Board of the University of Manitoba (H2015:124).

### Educational activity

Continuing Professional Development (CPD) activities range from passive, didactic large group presentations (e.g. educational meetings, conferences, seminars, lectures, etc.) to highly interactive learning methods, such as workshops, small groups and individualized training sessions [29]. Evidence has shown that lectures and symposia have a positive impact on physician knowledge and competence. In contrast, interactive CPD activities are the most effective at improving practice and patient health outcomes [30]. “*Obesity Intervention for Front-line Healthcare Providers*” combined educational formats (lecture in the morning session and an interactive workshop in the afternoon session) to optimize the learning process. More information about the educational activity can be found at http://umanitoba.ca/faculties/health_sciences/cca/obesity-day.html.

The morning sessions (7:45 a.m. -1:00 p.m.) “The evolving roles of healthcare practitioners in intervention of obesity” addressed the evolving roles of healthcare providers in preventing and managing obesity. First, the participants listened to a speaker share his personal life experiences as an obese individual, and the challenges and obstacles he faces in his daily life. This participation was followed by four presentations given by various healthcare providers. Topics covered included 1) the emerging crisis associated with obesity particularly in high-risk people, 2) evolving roles of healthcare practitioners in preventing and managing obesity, 3) new ideas for obesity prevention and management in the practices of healthcare and social service professionals, and 4) strategies for inter-disciplinary collaboration to promote the prevention of obesity and enhance the potential for reduced morbidity in people who are obese. Following the presentations, a Question and Answer panel took place. Just before lunch, a few case studies were briefly introduced, and participants were challenged to discuss over lunch how to work together to better intervene in the lives of the obesity-related cases in round tables with members of two or more professions. The objectives of this session were to: 1) Identify emerging crises associated with obesity, particularly in high-risk people in lower socio-economic populations; 2) Discuss the evolving roles of healthcare practitioners in intervention of obesity; 3) Implement novel, yet practical, ideas for obesity prevention and management in the practices of healthcare and social service professionals; 4) Collaborate with professionals outside of your discipline to promote prevention of obesity, and enhance the potential for reduced morbidity in people who are obese.

The afternoon sessions (1:30 p.m.-3:30 p.m.) “Patient counselling in obesity intervention” addressed patient counseling in obesity prevention and intervention. In the afternoon workshop, participants learned how to determine patients’ motivation and readiness for lifestyle modifications to achieve a healthy weight, and how to provide guidance in the form of simple steps to effective weight loss using standardized patients. The objectives of this session were: 1) Identify barriers to discussing weight loss with patients; 2) Discuss how to effectively begin a conversation about a patient’s weight; 3) Assess the patient’s readiness for life style modification to achieve a healthy weight; 4) Enhance proficiency in discussing obesity by practicing this skill set with standardized patients (patient actors).

### Survey

Authors could not find a comprehensive tool that would compile information about skills, attitudes and challenges toward obesity intervention among healthcare providers across disciplines in alignment to our study. Therefore, a survey was created based on a comprehensive list of perceived skill levels, professional attitudes and challenges toward obesity intervention compiled from existing literature [12–23] and the objectives of our learning activity. It included questions related to interprofessional practice which inquired about knowledge of who to refer patients to and management of obesity-related issues as part of their scope of practice.

The survey was informally piloted and refined by the planning committee; the members of the planning committee completed the survey individually, and subsequently discussed the questions as a team to improve clarity and alignment with the research objectives. Finally, the research associates (SM, HL) incorporated the feedback and made adjustments to the tool. The final instrument consisted of 3 components: 1) a unique identifier to link data from the three surveys created using single letters or numbers from the following questions: a) the last number of the person’s birth year, b) the first letter of the birth city, c) the first letter of the first legal name, d) the birth day, and e) the first letter of the biological mother’s first name; 2) basic demographics of the participants including gender, age, years of practice, and profession; 3) rankings of various aspects of obesity management (see below): a) perceived skills were rated from 1(low) to 3(high); b) professional attitudes and c) perceived challenges were rated using a 5 point Likert scale with the lowest number indicating “strongly disagree” and the highest number indicating “strongly agree” (Table 1. Survey questions).

**Table 1:** Survey questions

Perceived skills (1–3) 1 = Low to 3 = High	
My ability to assess weight status and associated risk factors	
My ability to address weight management and obesity issues with patients	
My ability to teach and motivate patients toward physical activity	
My ability to teach and motivate patients toward healthy eating practices	
My ability to use behavior modification techniques to make lifestyle changes in your patients	
My ability to deal with family issues around weight management	
Professional Attitudes (1–5) 1 = Strongly disagree 5 = Strongly agree	
I do not feel that obesity intervention is part of my scope of practice	
I believe that a clinician’s role is simply to raise the issue of obesity rather than intervene	
I do not have time to deal with the issue of obesity in my practice	
Obesity is too difficult an issue to tackle therefore I do not address it in my practice	
I feel overwhelmed by the issue of obesity	
I am not confident that any obesity intervention I attempt will make a difference	
I do not feel sufficiently educated or competent in obesity intervention strategies	
I do not know whom to refer patients in cases of obesity intervention	
I am not comfortable in discussing obesity with my patients	
I avoid bringing up the topic of obesity as I do not want to offend or jeopardize my relationship with my patients and/or their family members	
As a healthcare provider, I am extremely frustrated with the low success rate in managing obesity	
I feel that my patients will not be compliant and any obesity intervention efforts I attempt will have little impact, if any	
I do not feel the need to address obesity issues with my patients unless they look or act sick	
I fear that talking about obesity could do even more damage by leading my patient toward an eating disorder or other psychological problem	
Challenges (1–5) 1 = Strongly disagree 5 = Strongly agree	
Obesity intervention is not taught in my discipline’s curriculum before we enter practice	
There is limited professional training in this area (e.g. continuing professional development)	
Healthcare providers in my discipline are not adequately compensated for treating obesity	
There is a lack of appropriate referral options (e.g. dietitians or other related professionals)	
There is a lack of patient education materials regarding obesity to distribute to our patients	
Healthcare providers in my discipline need more guidance toward raising a sensitive issue such as obesity with our patients.	
Healthcare providers in my discipline need more guidance in motivational interviewing for behavior change related to obesity.	

### Analysis

Learning outcomes were assessed using Kirkpatrick’s model (level 2) which considers whether the participants acquired the intended knowledge, skills or attitudes based on their participation in the training or intervention collecting information both before and after the learning activity [31, 32].

Descriptive statistics were used to present the demographic information of the participants including gender, age range, profession, years of practice and self-described weight. The chi-square test and student t-test were used to analyze the differences in the demographic characteristics between participants who completed or did not complete pre- and post-activity surveys. Wilcoxon matched pairs test (*z*) were used to explore possible *changes* following exposure to the educational intervention immediately post- activity and at the six-month follow up. Effect sizes (*r*) =$z/\sqrt{n}\left(\mathrm{number}\ \mathrm{of}\ \mathrm{pairs}\right)$ were calculated (*r* ≤ 0.1 = small effect, *r* = 0.3–0.5 = moderate effect and *r* > 0.5 large effect). Correlation between practitioners’ characteristics and statistically significant pre-and post-activity changes in perceived skills and professional attitudes were explored. Correlation between practitioners’ characteristics and pre-activity changes in perceived challenges were also sought.

Statistical significance was accepted for *p*-values< 0.05. All analyses were performed using SPSS version 24.0 (IBM Corp., Armonk, NY, USA).

## Results

### Descriptive findings

**Fig. 1:**
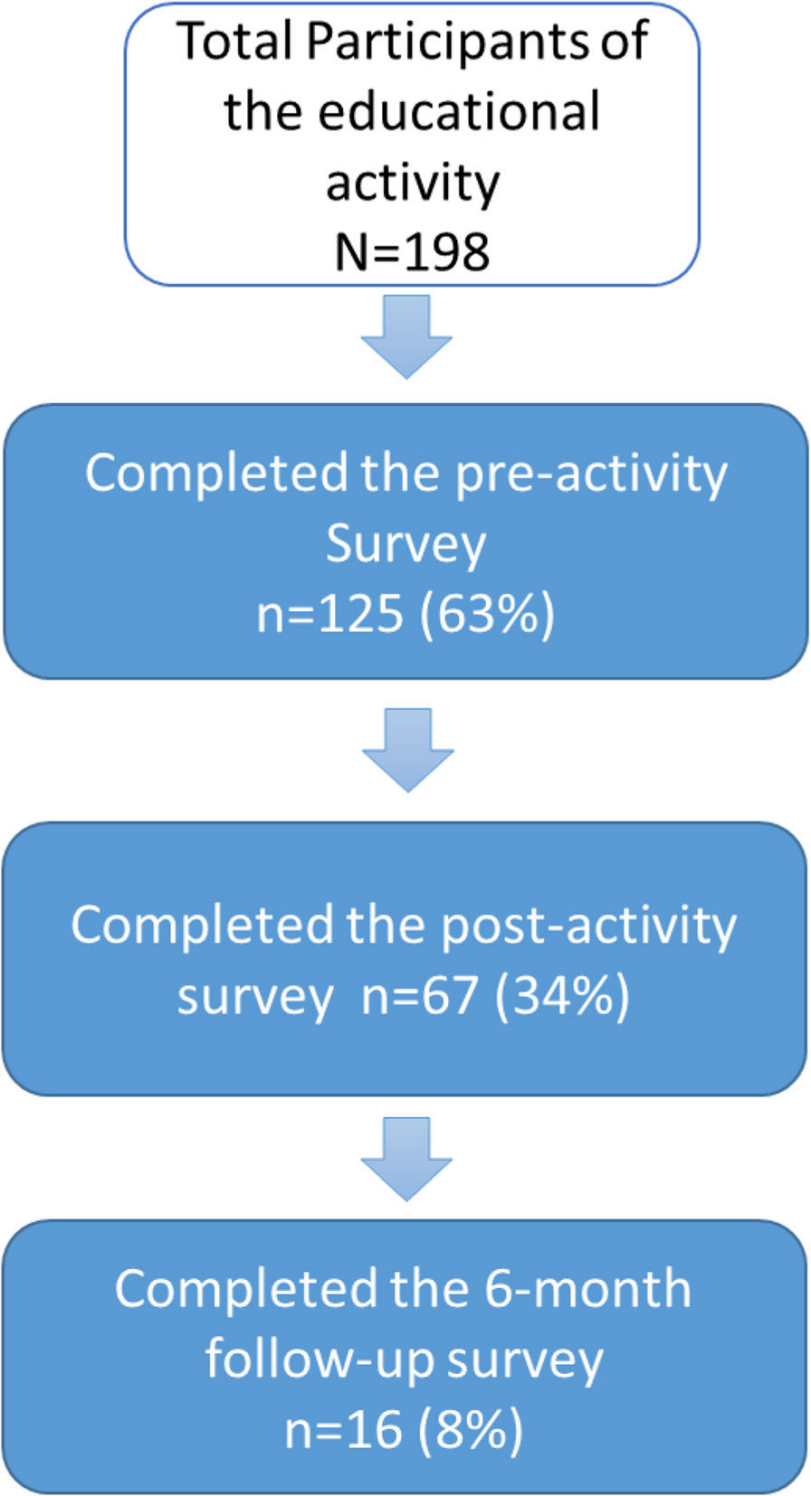
Participants’ flow chart

One hundred and twenty-five healthcare providers (63% of the total attendees) answered the pre-activity survey (Fig. 1). Of these, 68% were females, 54% were physicians, 45% had more than 21 years of clinical practice, and 69% considered themselves normal weight (Table 2). Fifty-four percent of the participants (34% of the total attendees) who answered the pre-survey also completed the post-activity survey immediately following the event; this group was statistically significantly younger (*p* = 0.02) and had fewer years of clinical practice (*p* = 0.03) than non post-activity responders. Thirteen percent of the initial participants (8% of the total attendants) responded to two post-activity surveys, one immediately after the educational activity and another at 6-month post-activity. No statistically significant differences in practitioner characteristics were found between participants who completed or did not complete the six-month follow-up survey (data not shown).

**Table 2:** Description of the participants

	All Participants who completed the Pre event survey (*n* = 125)	Post-event survey		
		Not completed (*n* = 58)	Completed (*n* = 67)	*p*
Gender				
Female, n (%)	85 (68)	33 (56.9)	52 (77.6)	0.07
Age Range				
20–29 years	13 (10.4)	2 (3.4)	11 (16.4)	**0.02**
30–39 years	25 (20.0)	8 (13.8)	17 (25.4)	
40–49 years	23 (18.4)	9 (15.5)	14 (20.9)	
50–59 years	27 (21.6)	17 (29.3)	10 (14.9)	
+ 60 years	35 (28.0)	20 (34.5)	15 (22.4)	
Type of healthcare provider				
Physician	67 (53.6)	35 (60.3)	32 (47.8)	0.10
Dietitian	15 (12.0)	6 (10.3)	9 (13.4)	
Nurse	9 (7.2)	4 (6.9)	5 (7.5)	
Physiotherapists	9 (7.2)	2 (3.4)	7 (10.4)	
Other	23 (18.4)	9 (15.7)	14 (21.3)	
Years in practice				
0–5	24 (19.2)	6 (10.3)	18 (26.9)	**0.03**
6–10	11 (8.8)	3 (5.2)	8 (11.9)	
11–15	15 (12.0)	6 (10.3)	9 (13.4)	
16–20	13 (10.4)	8 (13.8)	5 (7.5)	
+ 21	56 (44.8)	33 (56.9)	23 (34.4)	
How do you described your own weight?				
Underweight	0 (0)	0 (0)	0 (0)	0.29
Normal	86 (68.8)	42 (72.4)	44 (65.7)	
Overweight	34 (27.2)	13 (22.4)	21 (31.3)	

Percentages in some categories might not added to 100% due to some missing responses. Significant values **boldfaced** (*p* < 0.05).

### Perceived skills

Statistically significant post-activity increases in perceived skills were found (Table 3). Participants reported increases in their ability to assess weight status and associated risk factors (*r* = 0.25, *p* = 0.04), to address weight management issues with patients (*r* = 0.52, *p* < 0.001), to teach/motivate patients toward physical activity (*r* = 0.46, *p* < 0.001) and healthy eating practices (*r* = 0.42, *p* < 0.001), and to use behaviour modification techniques (*r* = 0.47, *p* < 0.001). A significant increase in practitioners’ ability to deal with family issues around weight management was found post-activity (*r* = 0.58, *p* < 0.001) and remained after 6 months (*r* = 0.53, *p* = 0.03).

**Table 3:** Changes in perceived skills, professional attitudes and perceived challenges after obesity day

	Pre-Post event				Pre-6 month			
	*n* = 67				*n* = 16			
Perceived skills, professional attitudes and challenges	Descriptive		Wilcoxon matched pairs test		Descriptive		Wilcoxon matched pairs test	
Perceived skills (1–3) 1 = Low to 3 = High	Pre Mean (SD)	Post Mean (SD)	*Z*	*p*	Pre Mean (SD)	6 month Post Mean (SD)	*Z*	*p*
My ability to assess weight status and associated risk factors	2.35 (0.6)	2.49 (0.5)	**−2.07**	**0.04**	2.62 (0.5)	2.43 (0.7)	−1.00	0.32
My ability to address weight management and obesity issues with patients	1.94 (0.6)	2.31 (0.5)	**−4.23**	**< 0.001**	1.93 (0.5)	2.19 (0.7)	−1.03	0.31
My ability to teach and motivate patients toward physical activity	1.88 (0.5)	2.24 (0.5)	**−3.80**	**< 0.001**	2.00 (0.5)	2.27 (0.7)	−1.27	0.21
My ability to teach and motivate patients toward healthy eating practices	1.98 (0.6)	2.27 (0.6)	**− 3.40**	**0.001**	2.00 (0.7)	2.25 (0.6)	−1.27	0.21
My ability to use behavior modification techniques to make lifestyle changes in your patients	1.76 (0.6)	2.09 (0.6)	**−3.88**	**< 0.001**	1.68 (0.7)	1.88 ((0.8)	−1.34	0.18
My ability to deal with family issues around weight management	1.52 (0.6)	1.96 (0.5)	**−4.77**	**< 0.001**	1.37 (0.5)	1.81 (0.6)	**−2.11**	**0.03**
Professional Attitudes (1–5) 1 = Strongly disagree 5 = Strongly agree								
I do not feel that obesity intervention is part of my scope of practice	1.74 (1.0)	1.52 (0.9)	−1.52	0.13	1.75 (0.8)	1.64 (0.9)	−1.00	0.32
I believe that a clinician’s role is simply to raise the issue of obesity rather than intervene	1.70 (0.7)	1.56 (0.7)	−1.60	0.11	1.75 (0.5)	1.71 (0.9)	−0.11	0.91
I do not have time to deal with the issue of obesity in my practice	2.26 (1.1)	2.17 (1.0)	− 0.73	0.46	2.07 (1.0)	2.07 (1.2)	− 0.33	0.74
Obesity is too difficult an issue to tackle therefore I do not address it in my practice	1.80 (0.7)	1.85 (0.8)	− 0.22	0.83	1.75 (0.8)	1.71 (0.9)	−0.45	0.66
I feel overwhelmed by the issue of obesity	2.84 (1.1)	2.75 (1.1)	−0.75	0.45	3.18 (1.1)	2.78 (1.1)	−1.44	0.15
I am not confident that any obesity intervention I attempt will make a difference	2.37 (0.9)	2.11 (0.7)	−1.67	0.09	2.56 (1.1)	2.29 (0.9)	−1.10	0.27
I do not feel sufficiently educated or competent in obesity intervention strategies	2.90 (1.0)	2.17 (0.9)	**−4.41**	**< 0.001**	3.00 (1.0)	2.36 (1.2)	**−2.07**	**0.04**
I do not know whom to refer patients in cases of obesity intervention	3.04 (1.2)	2.36 (1.0)	**−3.88**	**< 0.001**	3.12 (1.2)	2.43 (1.3)	**−2.43**	**0.02**
I am not comfortable in discussing obesity with my patients	2.32 (1.0)	1.83 (0.8)	**−3.76**	**< 0.001**	2.37 (1.2)	1.79 (1.0)	**−2.17**	**0.03**
I avoid bringing up the topic of obesity as I do not want to offend or jeopardize my relationship with my patients and/or their family members	2.27 (1.0)	1.97 (0.8)	**−2.86**	**< 0.01**	2.50 (1.1)	1.92 (0.9)	**−2.31**	**0.02**
As a healthcare provider, I am extremely frustrated with the low success rate in managing obesity	3.66 (0.9)	3.42 (0.9)	**−2.18**	**0.03**	3.75 (0.7)	3.28 (0.9)	**−2.13**	**0.03**
I feel that my patients will not be compliant and any obesity intervention efforts I attempt will have little impact, if any	2.59 (1.0)	2.40 (0.9)	−1.53	0.13	2.50 (1.0)	2.29 (0.9)	−1.13	0.23
I do not feel the need to address obesity issues with my patients unless they look or act sick	1.89 (0.8)	1.72 (0.7)	−1.66	0.09	2.12 (1.03)	1.86 (1.0)	−0.83	0.41
I fear that talking about obesity could do even more damage by leading my patient toward an eating disorder or other psychological problem	1.95 (0.8)	1.91 (0.8)	−0.36	0.72	2.18 (0.9)	2.14 (1.3)	−0.37	0.71
Challenges (1–5)^a^ 1 = Strongly disagree 5 = Strongly agree								
Obesity intervention is not taught in my discipline’s curriculum before we enter practice	3.10 (1.3)	–	–	–	3.06 (1.4)	2.92 (1.3)	0.00	1.00
There is limited professional training in this area (e.g. continuing professional development)	3.65 (1.0)	–	–	–	4.06 (0.9)	3.79 (1.1)	−1.41	0.16
Healthcare providers in my discipline are not adequately compensated for treating obesity	3.46 (0.9)	–	–	–	3.68 (1.1)	3.36 (1.3)	−1.63	0.10
There is a lack of appropriate referral options (e.g. dietitians or other related professionals)	3.46 (1.0)	–	–	–	3.68 (1.1)	3.57 (1.3)	−0.58	0.56
There is a lack of patient education materials regarding obesity to distribute to our patients	3.55 (1.0)	–	–	–	4.00 (0.8)	3.64 (1.2)	−1.29	0.12
Healthcare providers in my discipline need more guidance toward raising a sensitive issue such as obesity with our patients.	4.05 (0.8)	–	–	–	4.06 (0.9)	3.57 (1.3)	−1.47	0.14
Healthcare providers in my discipline need more guidance in motivational interviewing for behavior change related to obesity.	4.14 (0.7)	–	–	–	4.00 (0.9)	3.57 (1.2)	−1.38	0.17

Wilcoxon matched pairs test (z) Asymp Sig (2-tailed) p-value. *SD* Standard Deviation. ^a^Challenges were included only in the pre-event and six-month surveys. Significant values **boldfaced** (*p* < 0.05).

Correlations indicated that the increases in their ability to assess weight status and associated risk factors was larger among younger practitioners (*r* = 0.28, *p* = 0.02) and healthcare professionals other than physicians (*r* = 0.25, *p* = 0.04). Greater increases in their ability to address weight management issues with patients occurred among male practitioners (*r* = 0.25, *p* = 0.04) (Table 4).

**Table 4:** Correlation of practitioners’ characteristics with changes in perceived skills and professional attitudes after the obesity day (pre-post activity surveys)

	Practitioners characteristics			
	Gender^1^	Age Range	Physicians vs other HCP^2^	Years of practice
Change in Perceived skills Positive values indicate increase in perceived skills				
My ability to assess weight status and associated risk factors	–	*r* = −0.28	*r* = − 0.25	–
		*p* = 0.02	*p* = 0.04	
My ability to address weight management/obesity issues with patients	*r* = − 0.25	–	–	–
	*p* = 0.04			
My ability to teach and motivate patients toward physical activity	–	–	–	–
My ability to teach and motivate patients toward healthy eating practices	–	–	–	–
My ability to use behavior modification techniques to make lifestyle changes in your patients	–	–	–	–
My ability to deal with family issues around weight management	–	–	–	–
Change in Professional Attitudes Negative values indicate improvement in professional attitudes				
I do not feel sufficiently educated or competent in obesity intervention strategies	–	–	–	–
I do not know whom to refer patients in cases of obesity intervention	–	*r* = 0.36	*r* = 0.28	*r* = 0.32
		*p* = 0.004	*p* = 0.02	*p* = 0.01
I am not comfortable in discussing obesity with my patients	–	–	–	–
I avoid bringing up the topic of obesity as I do not want to offend or jeopardize my relationship with my patients and/or their family members	*r* = 0.26	–	–	–
	*p* = 0.03			
As a healthcare provider, I am extremely frustrated with the low success rate in managing obesity	–	–	–	–

Spearman’s correlation tests. This table included only changes that were statistically significant between pre and post-event (*n* = 67 participant) based on Table 2. Males^1^ and other HCP^2^ as reference. HCP=Healthcare providers. Variables as presented in Table 1

### Professional attitudes

The post-activity survey showed that practitioners learned to whom to refer patients for obesity interventions (*r* = 0.47, *p* < 0.01), felt more educated/competent in obesity intervention strategies (*r* = 0.54, *p* < 0.001), were more comfortable discussing obesity with their patients (*r* = 0.46, *p* < 0.001), were less likely to avoid the topic of obesity in order to not offend patients or jeopardize their relationships with patients and/or family members (*r* = 0.35, *p* = 0.004), and were less frustrated with the low success rate in managing obesity (*r* = 0.27, *p* = 0.03) (Table 3). Those changes remained six-month after the intervention showing stronger effect sizes (all *r* ≥ 0.52).

Improved knowledge to whom to refer patients in cases of obesity intervention correlated with younger practitioner age (*r* = 0.36, *p* < 0.001), healthcare professionals other than physicians (*r* = 0.28, *p* = 0.02) and fewer years of professional practice (*r* = 0.32, *p* = 0.01) (Table 4). Greater improvement in avoidance of the topic of obesity was correlated with male gender (*r* = 0.26, *p* = 0.03).

### Perceived challenges

Using pre-activity responses, the two main perceived challenges identified indicated that healthcare providers felt they need more guidance 1) towards raising obesity issues with their patients, and 2) in motivational interviewing for behaviour changes related to obesity. Older providers (*r* = 0.33, *n* = 122, *p* < 0.01) with more years of practice (*r* = 0.36, *n* = 122, *p* < 0.01) reported that obesity intervention was not taught in their curriculum before they entered practice. Physicians expressed that there is a lack of referral options in this area (*r* = 0.21, *n* = 121, *p* = 0.02) and were less likely to think that there is a lack of guidance toward raising a sensitive issue such as obesity with their patients compared with other healthcare providers (*r* = − 0.21, *n* = 122, *p* = 0.02). Questions related to the perceived challenges were included only in the pre-activity and six-month follow-up surveys. No statistically significant changes in perceived challenges were found six-months after the educational activity.

## Discussion

Outcomes of the present study showed that this interprofessional educational activity contributed to the improvement of professional skills and attitudes of front-line healthcare providers caring for those who are obese or at risk for obesity. In addition, a small sub-analysis of data from practitioners who participated in the 6-month follow-up survey suggested that significant effects remained over time. Primary healthcare providers play an important role in the prevention and management of obesity [33]. Therefore, it is important to offer them educational activities and resources to help them approach this growing healthcare challenge from an interprofessional approach to improve obesity outcomes. [25] The positive results found suggest that this interprofessional educational activity, which aligned with knowledge and training needs identified by healthcare providers in previous studies, [12–23] could be used to guide future educational interventions in the field of obesity.

From an interprofessional perspective it is important to highlight that healthcare providers reported increased knowledge to whom to refer patients in case of obesity intervention, which suggested that they learned about different roles of healthcare professionals in the management of obesity. No significant changes were found in regards of the question “I do not feel that obesity intervention is part of my scope of practice”, however, ratings were already positive at baseline.

Improvement in perceived skills and attitudes in obesity management were found mainly among males and, younger practitioners, with fewer years of professional practice and healthcare professionals other than physicians. The results indicate that healthcare providers with those characteristics might benefit the most from education in obesity prevention and intervention, which further reinforces the value of this learning activity. Previous studies reported that practitioners who were females [34], older providers [35] and primary care physicians were already highly inclined to offer weight lost counseling [36]. Nevertheless, these results should be interpreted with caution as the participants who responded to the post-activity survey were significantly younger and had fewer years of practice, which could cause an over representation of participants with these characteristics and thereby influencing this association.

In the six-month follow-up survey, no change was found in perceived challenges compared with the data collected in this category in the pre-activity survey. It is important to note that extensive resources were offered to course participants in obesity intervention and referral of at risk people. However, these resources did not address certain challenges identify by participant such as lack of obesity counseling or presence of obesity management in academic curricula. The lack of significant change in perceived challenges potentially modifiable with the educational activity could be influenced by the low statistical power related to the small number of participants in the 6-month follow-up survey.

Older physicians with many years of practice reported that obesity intervention was not taught in their medical school education and training. It was not until 2015 that the Canadian Medical Association acknowledged obesity as a chronic disease that required enhanced research, treatment and prevention strategies. In addition, healthcare practitioners agreed that professional training in obesity intervention (including continuing education) is very limited and there is a lack of guidance in dealing with this issue.

The growing epidemic of obesity, in addition to challenges around knowledge and training needs identified by healthcare providers in previous studies, [12–23] highlights the importance of developing and operationalizing continuing education programming that targets obesity prevention and intervention for healthcare providers across disciplines. The positive results found in the present study suggest that this interprofessional educational activity could be used to guide prospective educational interventions in the field of obesity. This has a great value considering that obesity management represents a special challenge and benefits from a collaborative interprofessional approach [25, 37]. Future research should be directed at exploring the long-term effect of this educational programming, possibly by looking at patient outcomes and quality indicators of care.

### Limitations and strengths

Some limitations of the present study should be considered. First, the post-activity improvements in perceived skills and professionals’ attitudes among practitioners might be attributed to the Hawthorne effect, defined as an alteration of behaviour in subjects of a study due to their awareness of being observed [38, 39]. However, despite the small number of participants that could have contributed to a potential loss of statistical power, some significant gains remained 6 months after the educational activity supporting the positive effect of the intervention. Second, participants who completed both pre and post-presentation surveys were statistically significantly younger and had fewer years of clinical practice than no post-presentation responders which could have influenced the results. Further, only 13% of pre-activity participants completed post-activity surveys immediately after the educational activity and at 6 months post-activity, which suggest that follow-up results should be interpreted with caution. Limited participation in surveys constitutes a challenge for data collection in CPD research, which may affect the generalizability of the findings. Future studies should aim to explore the mid- and long-term effect of this educational activity using a larger number of participants. Third, it is possible that participants taking part in this educational activity had a particular interest in the topic that could affect the results. Because healthcare providers often choose to attend continuing education programs that interest them, interest bias may have been introduced. Forth, the survey was not formally piloted. However, the instrument was created, discussed and refined by twelve professionals from various disciplines and two research associates. The main strength of the present study was the interdisciplinary nature of the learning activity targeted to practicing health care professionals. Previous studies have attempted to evaluate interprofessional education during professional training [40, 41]. However, to the best of our knowledge, no study had evaluated the effect of an interprofessional obesity-related CPD activity on professional skills and attitudes of healthcare professionals from diverse disciplines. In addition, since obesity affects all ages, the overall content of the educational activity and the survey used in this study pertains to the treatment of obesity in all age groups; this is different from previous studies that have limited research of obesity treatment to a specific age group (i.e. children, adolescents) [18, 42]. Results of this study provides insight into the professional skills, attitudes, and perceived challenges among healthcare providers regarding obesity intervention in the general population.

## Conclusion

Results of this study show that this interprofessional learning activity contributed to the improvement of professional skills and attitudes of front-line healthcare providers caring for those who are obese or at risk for obesity. The positive results of this interprofessional learning activity aligns with the training needs identified by healthcare practitioners in previous studies, and suggests that this design and content could be used to guide future educational programming in the care of obese people.
